# Long Non-coding RNAs Rian and Miat Mediate Myofibroblast Formation in Kidney Fibrosis

**DOI:** 10.3389/fphar.2019.00215

**Published:** 2019-03-11

**Authors:** Roel Bijkerk, Yu Wah Au, Wendy Stam, Jacques M. G. J. Duijs, Angela Koudijs, Ellen Lievers, Ton J. Rabelink, Anton Jan van Zonneveld

**Affiliations:** Department of Internal Medicine (Nephrology) and the Einthoven Laboratory for Experimental Vascular and Regenerative Medicine, Leiden University Medical Center, Leiden, Netherlands

**Keywords:** long non-coding RNA, kidney fibrosis, myofibroblast, pericyte, Miat, Rian, microRNA, RNA therapeutics

## Abstract

There is an increasing prevalence of chronic kidney disease (CKD), which associates with the development of interstitial fibrosis. Pericytes (perivascular fibroblasts) provide a major source of α-SMA-positive myofibroblasts that are responsible for the excessive deposition of extracellular matrix. In order to identify pericyte long non-coding RNAs (lncRNAs) that could serve as a target to decrease myofibroblast formation and counteract the progression of kidney fibrosis we employed two models of experimental kidney injury, one focused on kidney fibrosis (unilateral ureteral obstruction; UUO), and one focused on acute kidney injury that yields kidney fibrosis in the longer term (unilateral ischemia-reperfusion injury; IRI). This was performed in FoxD1-GC;tdTomato stromal cell reporter mice that allowed pericyte fate tracing. Tomato red-positive FoxD1-derivative cells of control and injured kidneys were FACS-sorted and used for lncRNA and mRNA profiling yielding a distinctive transcriptional signature of pericytes and myofibroblasts with 244 and 586 differentially expressed lncRNAs (>twofold, *P* < 0.05), in the UUO and IRI models, respectively. Next, we selected two differentially expressed and conserved lncRNAs, Rian (RNA imprinted and accumulated in nucleus) and Miat (Myocardial infarction associated transcript), and explored their potential regulatory role in myofibroblast formation through knockdown of their function with gapmers. While Miat was upregulated in myofibroblasts of UUO and IRI in mice, gapmer silencing of Miat attenuated myofibroblast formation as evidenced by decreased expression of *α-SMA*, *col1α1*, *SMAD2*, and *SMAD3*, as well as decreased α-SMA and pro-collagen-1α1 protein levels. In contrast, silencing Rian, which was found to be downregulated in kidney myofibroblast after IRI and UUO, resulted in increased myofibroblast formation. In addition, we found microRNAs that were previously linked to Miat (miR-150) and Rian (14q32 miRNA cluster), to be dysregulated in the FoxD1-derivative cells, suggesting a possible interaction between miRNAs and these lncRNAs in myofibroblast formation. Taken together, lncRNAs play a regulatory role in myofibroblast formation, possibly through interacting with miRNA regulation, implicating that understanding their biology and their modulation may have the potential to counteract the development of renal fibrosis.

## Introduction

Chronic kidney disease (CKD) has an estimated worldwide prevalence of about 8% (Hallan et al., 2009; Bruck et al., 2016) and, due to the increasing prevalence of non-communicable diseases such as diabetes and hypertension, the numbers of patients are ever increasing (United States Renal Data System’s Annual Data Report, 2010). Besides its high morbidity, CKD is a leading cause of death due to premature cardiovascular disease (Levey and Coresh, 2012). Irrespective of the etiology, the common pathway in the pathology of CKD involves the development of tubulointerstitial fibrosis characterized by myofibroblast proliferation and subsequent excessive extracellular matrix accumulation (Levey and Coresh, 2012). It has been previously demonstrated that pericytes (or perivascular fibroblasts), are the major source of myofibroblasts in renal fibrosis (Humphreys et al., 2010). As such, a better understanding of the molecular mechanisms that drive myofibroblasts formation may yield therapeutic targets aimed at counteracting the progression of CKD.

Recent work has established that long non-coding RNAs (lncRNA) function as novel critical transcriptional and post-transcriptional regulators of gene expression. lncRNAs impact numerous key biological processes, including epigenetic regulation, alternative splicing, protein synthesis, and cell cycle control. However, a role for lncRNAs in the (patho)physiology of kidney fibrosis is only recently emerging. For example, lncRNA Erbb4-IR was found to promote renal fibrosis by targeting miR-29b (Sun et al., 2018) while other studies demonstrated that inhibition of Linc00963 and H19 were protective against renal fibrosis (Xie et al., 2016; Chen et al., 2018).

In the current study we aimed to identify lncRNAs that are dysregulated specifically in pericytes in renal fibrosis in the process of myofibroblast formation and to determine whether the modulation of lncRNA expression levels can influence this formation. To that end, we used a FoxD1-GC;tdTomato mouse model to genetically label pericytes and its myofibroblast-derivatives and subsequently isolated them through fluorescence-activated cell sorter (FACS)-sorting from healthy kidneys and injured kidneys that were exposed to unilateral ureteral obstruction (UUO) or ischemia reperfusion injury (IRI). Following lncRNA profiling of the isolated cells, we found a signature of differentially expressed lncRNAs and show that specific lncRNAs, Miat (Myocardial infarction associated transcript, also known as Gomafu), and Rian (RNA Imprinted and Accumulated in Nucleus), are part of a rate limiting post-transcriptional network that can potentially be targeted to counteract myofibroblast formation.

## Materials and Methods

### Animals

All animal experimental protocols were approved by the animal welfare committee of the veterinary authorities of the Leiden University Medical Center. Standard chow diet and drinking water were provided *ad libitum*. Eight week old male FoxD1-GC;tdTomato Red mice were used. For the unilateral IRI model, via an abdominal incision, the renal artery and vein were identified and unilaterally clamped for 45 min using surgical clamps (S&T, Neuhausen, Switzerland). The contralateral, unclamped, kidneys were used as control kidneys. After 2 days the mice were anesthetized and euthanized. The UUO model was performed through a left flank incision under general anesthesia. The ureter was identified and ligated twice at the level of the lower pole of the kidney with two separate silk ties. After 10 days the mice were anesthetized and euthanized.

### Cell Culture

The NIH3T3 cell line (mouse fibroblasts) was obtained from ATCC (American Type Culture Collection, Manassas, VA, United States), maintained in Dulbecco’s modified Eagle medium (Gibco/Invitrogen, Breda, Netherlands) supplemented with 10% fetal calf serum (Bio Whittaker/Cambrex, Verviers, Belgium) and 1×L-glutamine (Invitrogen) with a final concentration of 2 mM and was incubated at 37°C in 5% CO_2_. Cells were treated with 10 ng/mL TGF-β1 (R&D) for 48 h unless otherwise indicated. The cells were transfected at 60–75% confluence with 50-nM locked nucleic acid (LNA) GapmeR (Exiqon, Vedbaek, Denmark) targeting Miat or Rian using Lipofectamine 3000 (Life Technologies, Carlsbad, CA, United States) according to the manufacturer’s protocol.

### Gapmer Design

Gapmers for Miat and Rian were designed using the online available Antisense Gapmer Designer from Exiqon (Vedbaek, Denmark). The specific sequences of the gapmers were as follows (5′–3′): Miat_Gapmer_1: AGATGCAGGCGATTAG; Miat_Gapmer_2: TAGCACTTTGATTGAC; Rian_Gapmer_1: CAATCAATCCTGGAGC; Rian_Gapmer_2: CAATCAATCCTGGAGC; Negative control Gapmer: AACACGTCTATACGC.

### FACS Sorting

Kidneys of three individual mice were used per injury model and were mechanically dissociated and filtered through 100 and 40 μm filters. The obtained cell suspension was sorted using the FACSAria II (BD Biosciences, Franklin Lakes, NJ, United States). Cell debris was excluded using a FSC-A/SSC-A plot (gate 1), doublets were excluded using a SSC-W/ SSC-H plot (gate 2), and a FSC-W/FSC-H plot (gate 3) and the three gates were combined. Next, in the combined exclusion gate, cells were positively identified for their fluorescent tomato signal.

### lncRNA, mRNA, and miRNA Profiling

The profiling was performed by Arraystar Inc according to protocol. In brief, for microarray analysis, the Agilent Array platform was employed. The sample preparation and microarray hybridization were performed based on the manufacturer’s standard protocols with minor modifications. Briefly, mRNA was purified from total RNA after removal of rRNA (mRNA-ONLY^TM^ Eukaryotic mRNA Isolation Kit, Epicentre). Then, each sample was amplified and transcribed into fluorescent cRNA along the entire length of the transcripts without 3′ bias utilizing a mixture of oligo(dT) and random primers (Arraystar Flash RNA Labeling Kit, Arraystar). The labeled cRNAs were hybridized onto the Mouse lncRNA Array v3.0 (8 K × 60 K, containing 35,923 lncRNAs and 24,881 coding transcripts, Arraystar). After having washed the slides, the arrays were scanned by the Agilent Scanner G2505C.

Agilent Feature Extraction software (version 11.0.1.1) was used to analyze the acquired array images. Quantile normalization and subsequent data processing were performed using the GeneSpring GX v12.1 software package (Agilent Technologies). After quantile normalization of the raw data, lncRNAs and mRNA that at least 6 out of 12 samples have flags in Present or Marginal (“All Targets Value”) were chosen for further data analysis. Differentially expressed lncRNAs and mRNAs with statistical significance between the two groups were identified through Volcano Plot filtering. MicroRNA profiling was previously described (Bijkerk et al., 2016).

### Pathway Analysis

Pathway analyses were performed using Ingenuity Pathway Analysis (IPA) software. Bias corrected *z*-scores were determined with a *z*-score higher than 2 or lower than -2 being considered statistically significant.

### RNA Isolation and qRT-PCR Analysis

Total RNA was isolated from FACS sorted kidneys or cell cultures using Trizol reagent (Invitrogen). To quantify mRNA levels, 250 ng total RNA was reverse transcribed using Iscript (Bio-Rad) according to the manufacturers protocol. Quantitative PCR of target genes was done using SYBR Green Master Mix (Applied Biosystems). Used primer sequences of target genes were: *α-SMA* (sense) CGTGGCTATTCCTTCG TGAC; *α-SMA* (antisense): GCGTTCGTAGCTCTTCTCC; *Col1α1* (sense): TGACTGGAAGAGCGGAGAGT; *Col1α1* (antisense): GTTCGGGCTGATGTACCAGT; *Smad2* (sense): AAGCCATCACCACTCAGAATTG; *Smad2* (antisense): CACTGATCTACCGTATTTGCTGT; *Smad3* (sense): CACGCAGAACGTGAACACC; *Smad3* (antisense): GGCAGTAGATAACGTGAGGGA; Rian (sense): CTGTTGTGCCCTCCCTGGATG; Rian (antisense): CCAGCTAGGCTGTGTAAATCATC; Miat(sense): CAGCCTCAAACCCAGGGC; Miat (antisense): CGCAGGACTGTTGTGCCA; *β-actin* (sense): AGGTCATCACTATTGGCAACGA; *β-actin* (antisense): CCAAGAAGGAAGGCTGGAAAA. Levels of expression were normalized to *β-actin* and quantified using the ΔΔ Ct method.

### Immunohistochemistry

Kidney were fixed in 4% PFA for 1 h at 4°C, cryopreserved in 20% sucrose, and frozen in liquid nitrogen. Five micrometers sections were stained for α-SMA using a mouse fluorescein isothiocyanate-conjugated antibody (Sigma). Images were made using a confocal microscope (Carl Zeiss, Sliedrecht, Netherlands).

### Statistical Analyses

Results are expressed as mean ± standard error of the mean (SEM), unless otherwise indicated. Statistical analysis was performed using Student’s *t*-test or one-way ANOVA. *P* < 0.05 were considered statistically significant.

## Results

### Myofibroblast Formation in Kidneys of UUO and IRI Mice

To follow the fate of pericytes and pericyte-derived myofibroblasts we used a genetic mouse model expressing a GFP-Cre (GC) fusion protein driven by the FoxD1 promotor (*FoxD1-GC*), as previously described (Humphreys et al., 2010; Bijkerk et al., 2016). In order to allow FACS sorting of fluorescently labeled pericyte-derived cells, we subsequently crossed the FoxD1-GC strain with the Tomato red reporter line. To study myofibroblast formation, we applied the UUO model that is known to induce severe kidney fibrosis and sacrificed the mice at 10 days. In addition, we included the unilateral IRI model as a milder (acute) kidney injury model but also known to develop kidney fibrosis and sacrificed the mice after 2 days.

**FIGURE 1:**
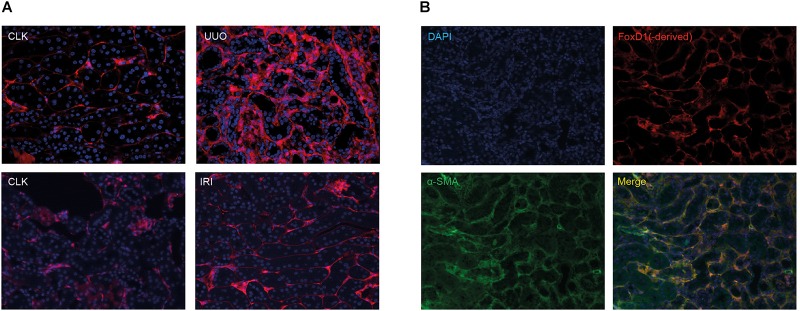
Tomato-positive fluorescent signal in healthy and injured kidneys in renal fibrosis models. **(A)** Fluorescent tomato signal of FoxD1(-derived) positive cells in FoxD1-Cre;tdTomato in the UUO model and the IRI model. CLK, contralateral (healthy) kidney. **(B)** Ten days after UUO, co-staining of tomato Red with α-SMA indicates the majority of myofibroblasts are derived from FoxD1-positive cells.

As shown in Figure 1A and confirming previous studies (Humphreys et al., 2010), 10 days after UUO we observed a marked expansion of tomato-positive pericyte-derived myofibroblasts in the interstitium of the fibrotic kidneys, while 2 days after unilateral IRI no clear expansion was observed. We confirmed myofibroblast formation of these tomato-positive cells in the UUO model by co-staining with α-SMA (Figure 1B). Sporadically we also observed double positive cells in the IRI model, already at 2 days after surgery (data not shown).

**FIGURE 2:**
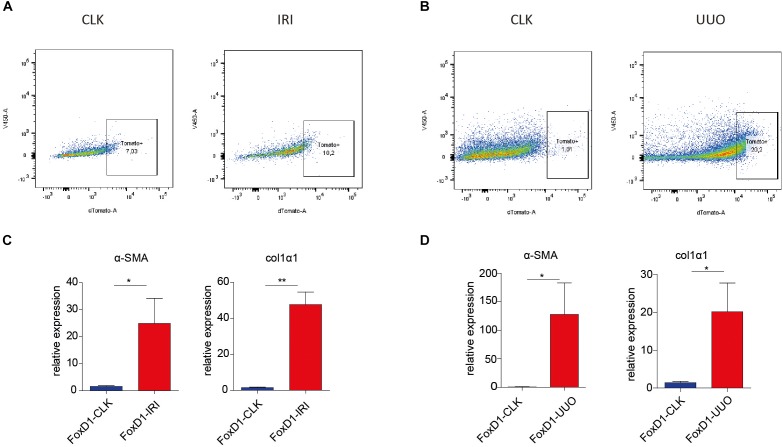
Fluorescence-activated cell sorter sorting of tomato-positive cells from healthy and injured kidneys in UUO and IRI model. **(A–D)** Representative FACS plots illustrating sorted cell populations (square boxes, number indicate cell fraction as percentage of total number of cells) in FoxD1-Cre;tdTomato mice in IRI **(A)** and UUO **(B)** models. **(C,D)** We confirmed upregulation in perivascular stromal cells in the IRI **(C)** and UUO **(D)** model of α-SMA and collagen-1α1 gene expression as determined by qRT-PCR on FACS sorted cells from FoxD1-tomato mice. CLK, contralateral (healthy) kidney. ^∗^*P* < 0.05, ^∗∗^*P* < 0.01. Data are represented as mean ± SEM. *N* = 3 (kidneys of three individual mice were used per injury model).

Next, FoxD1-derivative interstitial cells (tomato red positive) were isolated from mechanically dissociated cell suspensions of injured kidneys (UUO and IRI) and contralateral kidneys (CLK) using FACS sorting (Figure 2A,B). Quantitative PCR for myofibroblast markers on this isolated cell population confirmed their acquisition of a myofibroblast phenotype, as evidenced by increased expression of *α-SMA* and *col1α1* already 2 days after IRI or UUO (Figure 2C,D).

### lncRNAs Are Differentially Expressed During Myofibroblast Formation

**FIGURE 3:**
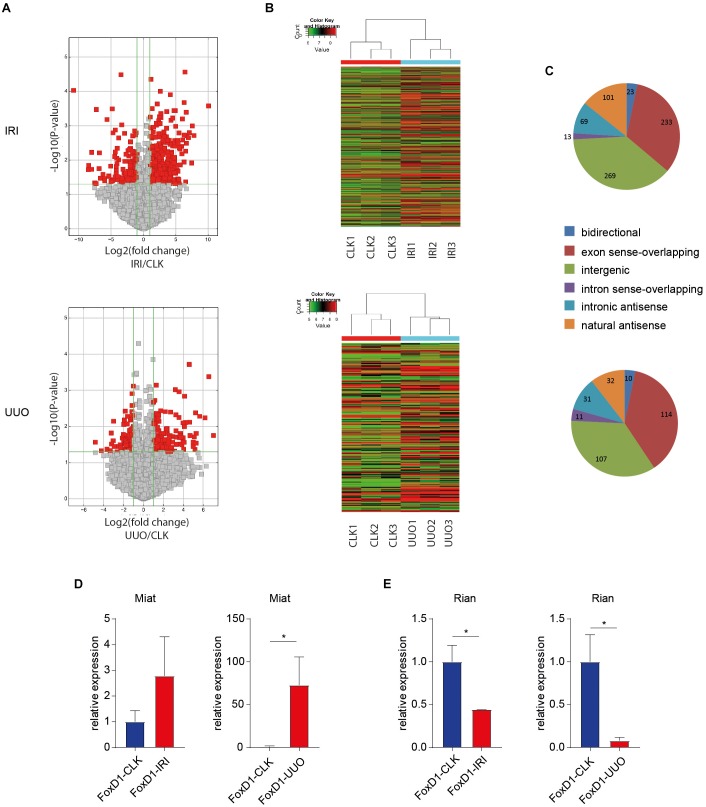
Differential lncRNA expression in IRI and UUO in pericytes and pericyte-derived myofibroblasts. **(A)** Volcano Plots are visualizing differential lncRNA expression between indicated conditions. The vertical lines correspond to 2.0-fold up and down, respectively, and the horizontal line represents a *p*-value of 0.05. So the red point in the plot represents the differentially expressed lncRNAs with statistical significance. **(B)** Hierarchical clustering shows a distinguishable lncRNA expression profiling among samples (*N* = 3 per condition). In UUO, we found 244 lncRNAs to be differentially expressed (>twofold, *P* < 0.05) in the FoxD1-derived perivascular cells. In IRI, we found 585 lncRNAs to be differentially expressed. **(C)** Classification of differentailly expressed lncRNAs. “sense_overlapping”: the lncRNA’s exon is overlapping a coding transcript exon on the same genomic strand; “intronic”: the lncRNA is overlapping the intron of a coding transcript on the same genomic strand; “natural antisense”: the lncRNA is transcribed from the antisense strand and overlapping with a coding transcript; “non-overlapping antisense”: the lncRNA is transcribed from the antisense strand without sharing overlapping exons; “bidirectional”: the lncRNA is oriented head to head to a coding transcript within 1000 bp; “intergenic”: there are no overlapping or bidirectional coding transcripts nearby the lncRNA **(D,E)** RT-qPCR on FACS sorted FoxD1-derivative interstitial cells indicated (a trend toward) increased Miat levels in IRI and UUO **(D)** and decreased Rian levels in IRI and UUO **(E)**. ^∗^*P* < 0.05, Data are represented as mean ± SEM. *N* = 3 [kidneys of three individual mice per injury model were FACS sorted followed by lncRNA profiling **(A,C)** and qPCR **(D,E)**].

To identify lncRNAs that are involved in the myofibroblastic response of pericytes in fibrotic kidney disease, we profiled lncRNAs from FoxD1-derivative interstitial cells that were isolated from injured and contralateral control kidneys. This provides a FoxD1-derivative interstitial cell-specific lncRNA profile within the *in vivo* renal injury setting. Figure 3A–C illustrates a clear differential lncRNA profile in pericyte-derived cells in diseased kidneys compared to healthy kidneys. In UUO, we found 244 lncRNAs to be differentially expressed (>twofold, *P* < 0.05) in the FoxD1-derived perivascular cells. In IRI, this was 586 lncRNAs (>twofold, *P* < 0.05). Supplementary Tables 1 (IRI) and 2 (UUO) contain all differen-tially expressed lncRNAs and characteristics.

### Associated Gene Expression Profiling and Pathway Analysis

To further dissect the molecular mechanisms that associate with these lncRNA profiles, we simultaneously performed whole genome expression profiling on these sorted pericyte-derived cells, that also allowed for subsequent pathway analyses. Supplementary Figure 1 illustrates a clear differential mRNA profile in pericyte-derived cells in diseased kidneys compared to healthy kidneys. In UUO, we found 192 mRNAs to be differentially expressed (>twofold, *P* < 0.05) in the FoxD1-derived perivascular cells. In IRI, this was 389 mRNAs (>twofold, *P* < 0.05). Supplementary Tables 3 (IRI) and 4 (UUO) contain all differentially expressed mRNAs and characteristics. Of note, we also determined pairs of differentially expressed lncRNA and associated coding genes (distance <300 kb), knowing that lncRNAs are typically coexpressed with their neighboring gene (Cabili et al., 2011). These data are indicated in Supplementary Tables 5 (IRI) and 6 (UUO).

Next, by using IPA, we analyzed affected biological pathways in the genome-wide differential gene expression datasets. Supplementary Figure 2 illustrates the top most affected canonical pathways. In the IRI model, in particular inflammatory pathways appear to be activated. In the UUO model, the cell cycle interestingly is amongst top regulated pathways, given that “cell cycle” was previously found to be a strongly regulated process in the development of kidney fibrosis (Grgic et al., 2014). Furthermore, confirming the fibrogenic nature of the injuries to the kidney, pathway analysis revealed top predicted potential upstream regulators, including the “hallmark genes” TGF-β1 and STAT3 (Bijkerk et al., 2016).

### Two Conserved lncRNAs, Rian, and Miat, Mediate Myofibroblast Formation

Next, we sought to investigate whether modulation of differentially expressed lncRNAs could abrogate myofibroblast formation. To that end, we selected two lncRNAs, namely Miat and Rian, based on the criteria that they were increased and decreased, respectively, in myofibroblasts in both IRI and UUO [Miat: *P* = 0.049, fold-change = 2.69 (IRI); *P* = 0.013, fold-change = 7.47 (UUO); Rian: *P* = 0.017, fold-change = -2.52 (IRI) and *P* = 0.025, fold-change = -2.62 (UUO)] and are conserved between mouse and humans. In total, 20 lncRNAs were differentially expressed in both IRI and UUO (Supplementary Table 7). Using RT-qPCR on the FACS sorted cells we validated differential Miat and Rian expression in FoxD1-derivative interstitial cells (Figure 3D,E).

**FIGURE 4:**
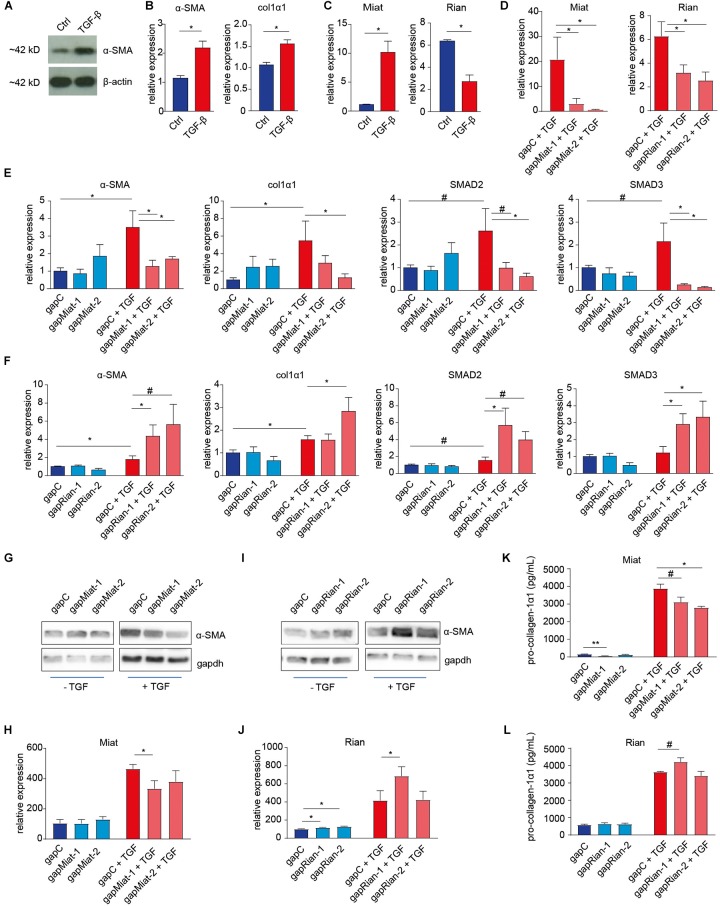
Involvement of lncRNAs Rian and Miat in myofibroblast formation. **(A,B)** Mouse fibroblasts (NIH3T3 cells) were used to model TGF-β induced myofibroblast formation, illustrated by the induction of α-SMA expression, determined by western blot **(A)** and *α-SMA* and collagen-1α1 gene expression **(B)**, determined by qRT-PCR. **(C)** Similar to the *in vivo* situation, Rian expression is decreased, while Miat expression in increased, upon TGF-β treatment of mouse fibroblasts. **(D)** Knockdown of Miat and Rian using gapmers upon treatment of 3T3 cells with TGF-β, as determined by qRT-PCR. *N* = 3 (three independent experiments) for panels **A–D**. **(E)** Knockdown of Miat using gapmers resulted in a decrease of *α-SMA*, *col1a1*, *SMAD2*, and *SMAD3* gene expression upon treatment of 3T3 cells with TGF-β, as determined by qRT-PCR. **(F)** Knockdown of Rian using gapmers increased *α-SMA*, *col1a1*, *SMAD2*, and *SMAD3* gene expression upon treatment of 3T3 cells with TGF-β, as determined by qRT-PCR. **(G,H)** Representative western blot images **(G)** and quantification **(H)** indicate that Miat knockdown results in decreased α-SMA levels. **(I,J)** Representative western blot images **(I)** and quantification **(J)** indicate that Rian knockdown results in increased α-SMA levels in non-stimulated cells, while only gapmer 1 increases α-SMA in the presence of TGF-β. *N* = 6 (six independent experiments) for panels **E–J**. **(K,L)** Pro-collagen-1α1 ELISA indicated Miat knockdown decreases pro-collagen levels **(K)**, while gapmer 1 for Rian increases pro-collagen-1α1 levels in the presence of TGF-β **(L)**. *N* = 3 (three independent experiments) for panels **K,L**. ^#^*P* < 0.01, ^∗^*P* < 0.05, ^∗∗^*P* < 0.01. Data are represented as mean ± SEM.

To investigate the impact of the selected lncRNAs on myofibroblast formation we used antisense “gapmers” to silence their expression in NIH3T3 mouse fibroblasts that, in response to TGF-β, undergo myofibroblast transition (Figure 4A–C). Using two different gapmers for both Miat and Rian, we first confirmed efficient knockdown by RT-qPCR (Figure 4D). This gapmer-mediated knock down of Miat inhibited myofibroblast formation as evidenced by decreased expression of *α-SMA*, *col1α1*, *SMAD2*, and *SMAD3* (Figure 4E). Conversely, knock down of Rian expression using two different gapmers resulted in increased myofibroblast formation and *α-SMA*, *col1α1*, *SMAD2*, and *SMAD3* gene expression (Figure 4F), recapitulating the *in vivo* association of lower Rian levels in fibrotic kidney derived myofibroblasts. Moreover, western blot and ELISA analyses further confirmed the inhibition of α-SMA (Figure 4G,H) and pro-collagen-1α1 protein expression (Figure 4K) upon Miat knockdown. While Rian gapmer 2 proved ineffective, Rian silencing with gapmer 1 resulted in increased levels of α-SMA (Figure 4I,J) and pro-collagen-1α1 (Figure 4L).

### Miat and Rian Associate With Altered MicroRNA Levels

Miat and Rian have both strongly been linked to microRNA function. For instance, increased Miat levels mediate vascular injury via sponging miR-150-5p (Yan et al., 2015) and associate with decreased miR-24 levels in cardiac fibrosis (Qu et al., 2017). On the other hand, Rian is part of the autoregulatory human 14q32 locus (Benetatos et al., 2013) (while in mice this is the analog 12qF1 locus) (Hagan et al., 2009), which harbors a large number of non-coding RNAs, including a cluster of miRNAs, that are well known for its function in vascular homeostasis (Welten et al., 2014; Aavik et al., 2015). When assessing the miRNA profile of FoxD1-derivative interstitial cells (Bijkerk et al., 2016) we demonstrated that while Miat was strongly upregulated in the myofibroblasts, no change was observed in the Miat related miR-24 level (data not shown). In contrast, we found miR-150 to be strongly increased in the FoxD1-derivative interstitial cells (Supplementary Figure 3C). Interestingly, concomitant with the downregulation of the 14q32-derived (mouse 12qF1) lncRNA Rian in the myofibroblast, 16 out of the 17 tested 14q32 derived miRNAs were upregulated (Supplementary Figures 3A,B), suggesting strong coordinate regulation of the 14q32 cluster derived non-coding RNAs in the progression of kidney fibrosis.

## Discussion

In this study, we demonstrated that the pericyte lncRNA expression profile is strongly altered upon myofibroblast formation in of kidney fibrosis. Silencing of two selected and strongly conserved lncRNAs, Miat and Rian, resulted in decreased and increased myofibroblast formation, respectively, illustrating that lncRNAs are important mediators of this process. As such, our data support the identification of a role for lncRNAs in the signaling networks that drive renal fibrosis by affecting the formation of myofibroblasts.

Tubulointerstitial fibrosis, as well as degeneration of the tubular epithelium is preceded by a loss of the capillary network (rarefaction) in the kidney (Long et al., 2012). This loss of the peritubular capillary network is directly correlated with the severity of fibrosis, and the extent of rarefaction has been found to predict the degree of interstitial damage as well as changes in the glomerular filtration rate in CKD patients (Seron et al., 1990; Choi et al., 2000). These findings indicate an early, rate-limiting role for microvascular destabilization/loss in the pathogenesis of fibrosis. Pericytes, that play a critical role in the stabilization of capillaries (Armulik et al., 2011) are also a major source of myofibroblasts, As such, it is intriguing that we identified two lncRNAs in these cells that have previously also been related to the maintenance of vascular integrity. First, Rian, a lncRNA located in the 14q32 cluster (in human, analogous to the mouse 12qF1 locus), is known to be co-regulated, in a methylation dependent manner, with the miRNAs from this cluster (Lehner et al., 2014) that are involved in inhibition of neovascularization (Welten et al., 2014). In keeping with this finding, we found 14q32-derived miRNAs to be consistently upregulated, suggesting a role for Rian in renal microvascular (dys)function. Moreover, this same miRNA cluster was recently demonstrated to be involved in the development of diabetic nephropathy through lncRNA-mediated regulation (Kato et al., 2016) of mainly miR-379, a microRNA that we also found to be increased in expression. Also the lncRNA Meg3 from this cluster has been shown to regulate the TGF-β pathway (Mondal et al., 2015). Important to note, the Irm transcript has been shown to partially overlap Rian in mouse (Hagan et al., 2009). Most likely, Irm represents a different isoform of the Rian gene^1^ (Gu et al., 2012), while it has been demonstrated that both transcripts exist and have different expression patterns (and functions) (Gu et al., 2012). As such, we cannot exclude a role for Irm in this study, although the region targeted by the gapmers lies outside the Irm region. Also, since in human and mice this locus, although similar, is located on different chromosomes, care is necessary with extrapolating mouse data to humans as they may be subject to different regulatory mechanisms. Furthermore, in contrast to mRNA levels, we did not observe increased α-SMA protein and pro-collagen-1α1 levels using the second gapmer for Rian. Although this might be just experimental variation, it is possible there is a regulatory role for the specific sequences targeted by the gapmers yielding the difference in effects. Another possibility is that different isoforms of Rian are targeted by the different gapmers, resulting in different outcomes, while also off-target effects cannot be excluded, although this is unlikely given the inclusion of a negative control gapmer. Nonetheless, we believe it seems likely that Rian and the components of the 14q32 cluster are crucial in the development of renal fibrosis and CKD.

Second, Miat was also previously described to regulate microvascular dysfunction, by functioning as a competing endogenous RNA (ceRNA) for miR-150 (Qu et al., 2017). This mechanism would provide a feedback loop with vascular endothelial growth factor (VEGF) and as such may well regulate endothelial cell survival and angiogenesis. A recent study described Miat to also function as a ceRNA for miR-132, which we previously demonstrated to mediate myofibroblast proliferation 10 days after UUO (Bijkerk et al., 2016). However, given the increase in Miat levels, this would suggest Miat should have anti-fibrotic effects via inhibiting miR-132 function, while here we find pro-fibrotic effects of Miat, similar to Miat function in the heart (Qu et al., 2017). These lncRNA-miRNA interactions therefore would need further investigation to clarify their exact roles and the dynamics of their expression. Furthermore, while we identified a role for Miat and Rian in (myo)fibroblasts, although NIH3T3 cells may not fully represent kidney-specific (myo)fibroblasts, it cannot be excluded they have additional functions in other cells that could impact the development of kidney fibrosis. For example, Miat also mediates high glucose-induced renal tubular epithelial cell injury (Zhou et al., 2015). Moreover, we identified several other lncRNA to be differentially regulated which could also play important roles in the development of kidney fibrosis, while comparing differentially expressed lncRNAs that are uniquely induced or repressed in either the IRI or the UUO model could possibly indicate IRI- or UUO-“specific” regulation and may represent an acute versus chronic response. Lastly, although pericytes most likely represent the major source of myofibroblasts, other sources have been described as well, including bone marrow derived cells (LeBleu et al., 2013; Buchtler et al., 2018; Kramann et al., 2018), endothelial cells (Zeisberg et al., 2008; LeBleu et al., 2013), and possibly some epithelial cells (LeBleu et al., 2013), that have not been included in our studies but may play an important role in the development of kidney fibrosis and may be regulated by various lncRNAs.

Taken together, our studies provided a comprehensive (post)transcriptional landscape of the transition of perivascular fibroblasts toward myofibroblasts in an *in vivo* injury setting. In addition, we demonstrated that modulating lncRNAs has the potential to counteract myofibroblast formation *in vitro* and therefore potentially the development of kidney fibrosis. As such, therapies aimed at modulating lncRNAs may represent novel potential treatments for CKD.

## Data Availability

The datasets generated for this study can be found in the Supplementary Files.

## Author Contributions

RB and AvZ conceived and designed the research. YA, WS, JD, AK, and EL performed the experiments. YA, JD, and RB analyzed the data. RB, TR, and AvZ interpreted the results of experiments. RB drafted the manuscript. All authors edited and revised the manuscript, and approved the final version of the manuscript.

## Conflict of Interest Statement

The authors declare that the research was conducted in the absence of any commercial or financial relationships that could be construed as a potential conflict of interest.

## Footnotes

1 http://lncrnadb.com/Rian/
