# Supplementary material for: Long Non-coding RNAs Rian and Miat Mediate Myofibroblast Formation in Kidney Fibrosis
Source: Front Pharmacol. 2019 Mar 11;10:215. doi: 10.3389/fphar.2019.00215 (PMC6421975; doi:10.3389/fphar.2019.00215)
Supplement: Supplementary file 5 [file Table_5.DOCX]

**Supplementary Table 5.** This table contains the differentially expressed lincRNA and associated coding gene pairs (distance < 300 kb) in the IRI model in FoxD1-tdTomato mice.

| **seqname** | **GeneSymbol** | **P-value - LncRNAs** | **Fold change - LncRNAs** | **NearbyGeneSymbol** | **P-value - mRNAs** | **Fold change - mRNAs** |
| --- | --- | --- | --- | --- | --- | --- |
| humanlincRNA0789+ | humanlincRNA0789 | 0,000362698 | 26,0089388 | Secisbp2l | 0,023630748 | 16,1345679 |
| uc008hww.1 | AK019949 | 0,000386751 | 12,0681001 | Rbm20 | 0,010764664 | -4,0902512 |
| ENSMUST00000181539 | 5830416P10Rik | 0,001011791 | 186,1745035 | Rbm20 | 0,010764664 | -4,0902512 |
| ENSMUST00000122590 | Gm25842 | 0,002614177 | 13,0688205 | Ryk | 0,00045907 | 15,480671 |
| uc007diu.1 | AK076808 | 0,004783893 | 4,321597 | Sft2d2 | 0,004115209 | 6,9116104 |
| ENSMUST00000131400 | Spi1 | 0,005126773 | 8,923802 | Slc39a13 | 0,004762497 | -75,4805833 |
| ENSMUST00000118913 | Gm14838 | 0,006092157 | 2,1835357 | Cdx4 | 0,002485937 | 178,7976089 |
| TCONS_00000514 | XLOC_001348 | 0,006928433 | 4,3774011 | Fam78b | 0,012506559 | 20,0630156 |
| TCONS_00021017 | XLOC_015982 | 0,010619421 | 3,3931895 | Lysmd1 | 0,015526671 | -2,4632777 |
| TCONS_00036235 | XLOC_027250 | 0,010746037 | 14,4727576 | Cdx4 | 0,002485937 | 178,7976089 |
| ENSMUST00000118094 | Olfr400-ps1 | 0,014356101 | 10,2908304 | Olfr401 | 0,013269931 | 13,6770065 |
| ENSMUST00000181571 | Gm26701 | 0,014904029 | -34,0726952 | Serpinb6c | 0,030346107 | -2,6572779 |
| mouselincRNA0813- | mouselincRNA0813 | 0,01548081 | 26,2727383 | Tle4 | 0,002788642 | 3,6637351 |
| ENSMUST00000119334 | Gm14182 | 0,020377135 | 16,3345915 | Gm11554 | 0,043560342 | 2,6840869 |
| uc009nwn.1 | AK076318 | 0,024070759 | 5,8479119 | Acta1 | 0,00307031 | -3,5590562 |
| uc009nwn.1 | AK076318 | 0,024070759 | 5,8479119 | Gas8 | 0,033919647 | 8,3861296 |
| ENSMUST00000126231 | 4930455G09Rik | 0,027044833 | 2,2333448 | Ctrc | 0,001099361 | -3,9946331 |
| uc007tup.1 | TCR-alpha chain | 0,027675387 | -2,1978383 | Abhd4 | 0,015072254 | 14,0259945 |
| ENSMUST00000174057 | Gm20527 | 0,03017762 | -2,6181449 | Gm9573 | 0,00335806 | 17,2009118 |
| TCONS_00017318 | XLOC_013140 | 0,030908382 | 6,8900351 | Mdk | 0,000887931 | 7,9775899 |
| ENSMUST00000181148 | C430039J16Rik | 0,031025322 | 3,3285734 | Enc1 | 0,007133538 | 15,5305746 |
| ENSMUST00000159838 | 4930448H16Rik | 0,032811653 | 2,7743824 | E130309D02Rik | 0,003259329 | 3,5647156 |
| AK041646 | AK041646 | 0,033755873 | 3,7402044 | Lef1 | 0,029658223 | 5,5536412 |
| ENSMUST00000120817 | Gm7212 | 0,034069707 | -66,140151 | Sash3 | 0,02586593 | -2,1569764 |
| ENSMUST00000121235 | Gm12318 | 0,03536647 | -4,6888923 | Inca1 | 0,011582481 | -18,3031497 |
| ENSMUST00000121235 | Gm12318 | 0,03536647 | -4,6888923 | Spag7 | 0,0027183 | 2,4484934 |
| ENSMUST00000140417 | Ido2 | 0,036394172 | -5,3401585 | Adam5 | 0,003231168 | 27,188661 |
| ENSMUST00000140417 | Ido2 | 0,036394172 | -5,3401585 | Adam5 | 0,002326381 | 29,0441996 |
| humanlincRNA1385- | humanlincRNA1385 | 0,039710046 | -63,5775507 | Tkt | 0,003532137 | -2,2440873 |
| AK149443 | AK149443 | 0,039972918 | 2,7626032 | Iglon5 | 0,006993972 | 22,5479099 |
| AK041575 | AK041575 | 0,044198987 | 2,0389893 | Tox | 0,032918045 | -4,5476934 |
| TCONS_00004448 | XLOC_004483 | 0,045126967 | 2,0133381 | Rnf43 | 0,02612726 | 2,2282547 |
| ENSMUST00000118809 | Olfr409-ps1 | 0,045411312 | 6,6317862 | Olfr401 | 0,013269931 | 13,6770065 |
