# Supplementary material for: Long Non-coding RNAs Rian and Miat Mediate Myofibroblast Formation in Kidney Fibrosis
Source: Front Pharmacol. 2019 Mar 11;10:215. doi: 10.3389/fphar.2019.00215 (PMC6421975; doi:10.3389/fphar.2019.00215)
Supplement: Supplementary file 6 [file Table_6.DOCX]

**Supplementary Table 6.** This table contains the differentially expressed lincRNA and associated coding gene pairs (distance < 300 kb) in the UUO model in FoxD1-tdTomato mice.

| **seqname** | **GeneSymbol** | **P-value - LncRNAs** | **Fold change - LncRNAs** | **NearbyGeneSymbol** | **P-value - mRNAs** | **Fold change - mRNAs** |
| --- | --- | --- | --- | --- | --- | --- |
| TCONS_00013580 | XLOC_010800 | 0,003767604 | -3,5403909 | Esp15 | 0,004438751 | 6,5368896 |
| TCONS_00013581 | XLOC_010800 | 0,006616419 | -3,7483996 | Esp15 | 0,004438751 | 6,5368896 |
| uc007coq.1 | AK155877 | 0,029411701 | 2,2859336 | Nfasc | 0,036795736 | 4,7451257 |
| AK004365 | AK004365 | 0,017804995 | 12,8370426 | Aif1l | 0,001541522 | 3,6062425 |
| AK132047 | AK132047 | 0,040044709 | 5,0067334 | Nfib | 0,037724395 | 2,2743845 |
