# Supplementary material for: Long Non-coding RNAs Rian and Miat Mediate Myofibroblast Formation in Kidney Fibrosis
Source: Front Pharmacol. 2019 Mar 11;10:215. doi: 10.3389/fphar.2019.00215 (PMC6421975; doi:10.3389/fphar.2019.00215)
Supplement: Supplementary file 7 [file Table_7.DOCX]

| **GeneSymbol** | **Seqname** | **Injury** | **P-value** | **Fold Change IRI or UUO divided by CLK** |
| --- | --- | --- | --- | --- |
| 1810062O18Rik | ENSMUST00000124940 | IRI | 0,0242 | 2,21 |
|  |  | UUO | 0,0318 | -2,99 |
| 4930449E01Rik | NR_045921 | IRI | 0,0126 | 7,60 |
|  |  | UUO | 0,0036 | 3,32 |
| AK018904 | AK018904 | IRI | 0,0251 | 9,95 |
|  |  | UUO | 0,0463 | -3,01 |
| AK036147 | AK036147 | IRI | 0,0065 | 61,18 |
|  |  | UUO | 0,0061 | 2,81 |
| AK079717 | AK079717 | IRI | 0,0126 | 9,19 |
|  |  | UUO | 0,0399 | 2,33 |
| AK133114 | AK133114 | IRI | 0,0209 | -5,18 |
|  |  | UUO | 0,0053 | -3,02 |
| Aqp7 | ENSMUST00000144201 | IRI | 0,0058 | 20,30 |
|  |  | UUO | 0,0039 | 4,53 |
| BC094917 | uc007tcy.1 | IRI | 0,0351 | 6,58 |
|  |  | UUO | 0,0042 | 37,30 |
| Crocc | ENSMUST00000151455 | IRI | 0,0384 | 2,51 |
|  |  | UUO | 0,0487 | 2,67 |
| Gm14104 | ENSMUST00000135990 | IRI | 0,0266 | -3,88 |
|  |  | UUO | 0,0283 | 3,21 |
| Gm20507 | ENSMUST00000163245 | IRI | 0,0106 | -3,22 |
|  |  | UUO | 0,0269 | 15,64 |
| Gm5421 | ENSMUST00000181698 | IRI | 0,0019 | 78,56 |
|  |  | UUO | 0,0361 | 2,62 |
| Gm6536 | ENSMUST00000171180 | IRI | 0,0016 | 135,38 |
|  |  | UUO | 0,0177 | 131,61 |
| Miat | NR_003718 | IRI | 0,0498 | 2,69 |
|  |  | UUO | 0,0135 | 7,47 |
| Pde7b | ENSMUST00000169016 | IRI | 0,0052 | 2,73 |
|  |  | UUO | 0,0288 | 2,30 |
| Plekhg5 | uc008vzg.1 | IRI | 0,0074 | 14,64 |
|  |  | UUO | 0,0131 | 9,68 |
| Rab40c | uc008bcn.1 | IRI | 0,0302 | -15,66 |
|  |  | UUO | 0,0002 | 24,24 |
| Rian | uc011ysu.1 | IRI | 0,0165 | -2,52 |
|  |  | UUO | 0,0253 | -2,62 |
| Sema3c | ENSMUST00000115271 | IRI | 0,0471 | 4,04 |
|  |  | UUO | 0,0465 | 2,27 |
| uc.292 | uc.292- | IRI | 0,0369 | -15,90 |
|  |  | UUO | 0,0052 | 50,18 |

**Supplementary Table 7.** LncRNAs that are differentially expressed in both IRI and UUO models.
