# Supplementary material for: Long Non-coding RNAs Rian and Miat Mediate Myofibroblast Formation in Kidney Fibrosis
Source: Front Pharmacol. 2019 Mar 11;10:215. doi: 10.3389/fphar.2019.00215 (PMC6421975; doi:10.3389/fphar.2019.00215)
Supplement: Supplementary file 8 [file Data_Sheet_1.PDF]

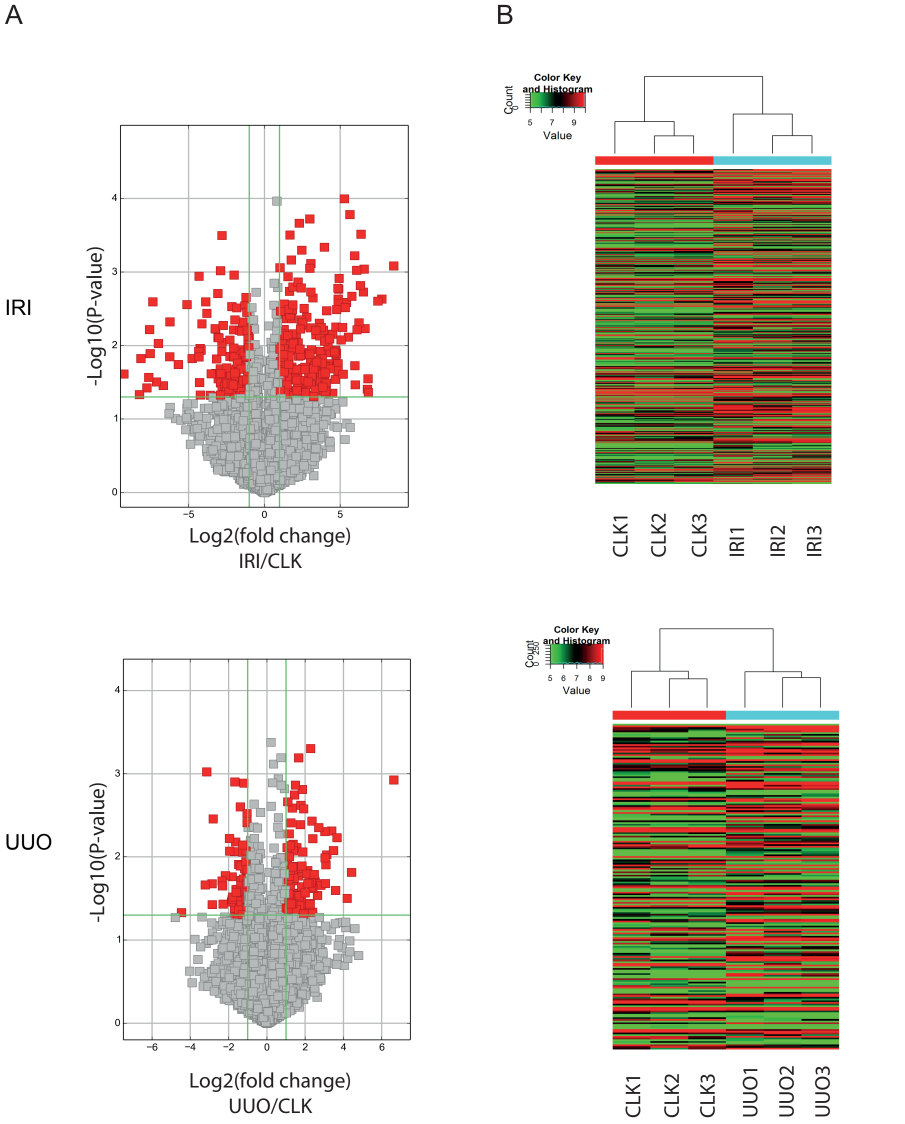

Supplementary Figure 1. Differential mRNA expression in IRI and UUO in pericytes and pericyte-derived myofibroblasts. (A) Volcano Plots are visualizing differential mRNA expression between indicated conditions. The vertical lines correspond to 2.0-fold up and down, respectively, and the horizontal line represents a p-value of 0.05. So the red point in the plot represents the differentially expressed mRNAs with statistical significance. (B) Hierarchical clustering shows a distinguishable mRNA expression profiling among samples (n=3 per condition). In UUO, we found 192 mRNAs to be differentially expressed (>2-fold, p<0.05) in the FoxD1-derived perivascular cells. In IRI, this is 389 mRNAs.
