# Supplementary material for: Long Non-coding RNAs Rian and Miat Mediate Myofibroblast Formation in Kidney Fibrosis
Source: Front Pharmacol. 2019 Mar 11;10:215. doi: 10.3389/fphar.2019.00215 (PMC6421975; doi:10.3389/fphar.2019.00215)
Supplement: Supplementary file 9 [file Data_Sheet_2.PDF]

A

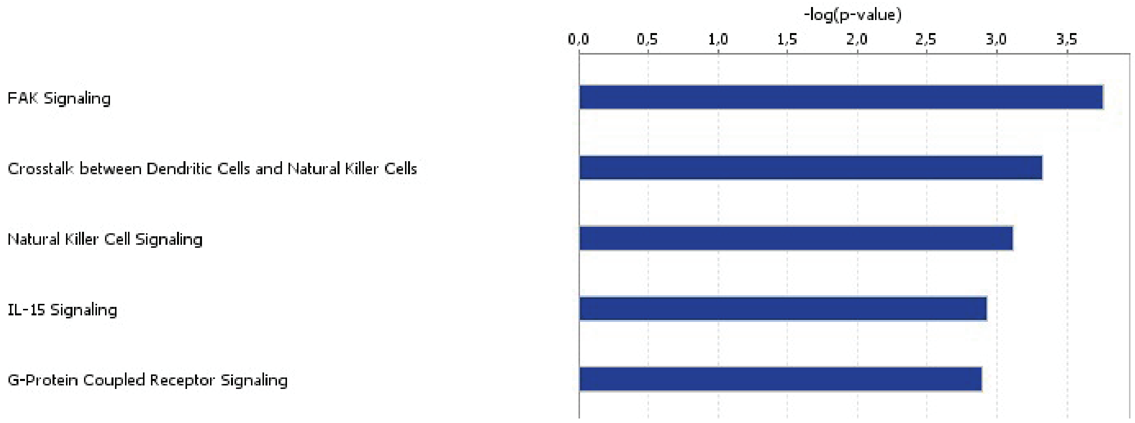

IRI

B

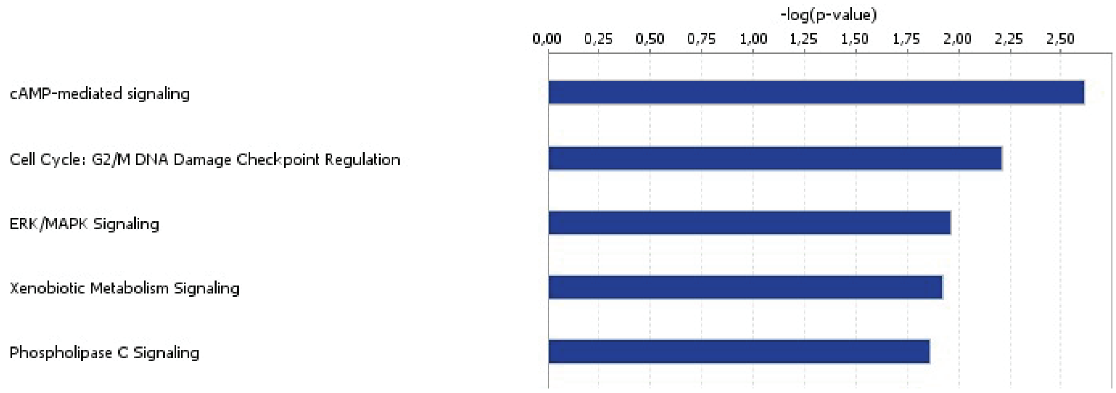

UUO

C

Upstream Regulator	*p-value of overlap*	*Predicted Activation State*	*Activation z-score*
**STAT3**	1,88E-02	Activated	2.254
**MITF**	2,16E-02	Activated	2.813
**EPO**	3,92E-02	Inhibited	-1,982
**TCF7L2**	4.33E-02	Activated	2.121
**CCL5**	9,67E-02	Activated	2.000

IRI

D

Upstream Regulator	*p-value of overlap*	*Predicted Activation State*	*Activation z-score*
**TGFB1**	3.56E-04	Activated	0.818
**CTNNB1**	5.56E-04	Inhibited	-0.200
**TCF7L2**	9.36E-03	Activated	1.134
**TNF**	9.86E-02	Activated	1.808
**NFKBIA**	9.95E-02	Activated	2.183

UUO

Supplementary Figure 2. Pathway analysis on differentialy expressed genes in perivascular stromal cells from FoxD1-tomato IRI and UUO models. (A-D) Differentially expressed genes (IRI or UUO versus healthy control kidney (contralateral kidney; CLK)), as determined by whole genome expression profiling of FACS sorted tomato-positive cells from the IRI and UUO model in FoxD1-tomato mice, were analyzed using Ingenuity Pathway Analysis software to identify potentially regulated canonical pathways (A-B) (top-ranked pathways based on p-value) and upstream regulators (C-D) (lower top-ranked regulators based on p-value). Identified upstream regulators are molecules that are known to affect the expression of several differentially expressed downstream genes as found in the profiling.
