# Supplementary material for: Long Non-coding RNAs Rian and Miat Mediate Myofibroblast Formation in Kidney Fibrosis
Source: Front Pharmacol. 2019 Mar 11;10:215. doi: 10.3389/fphar.2019.00215 (PMC6421975; doi:10.3389/fphar.2019.00215)
Supplement: Supplementary file 10 [file Data_Sheet_3.PDF]

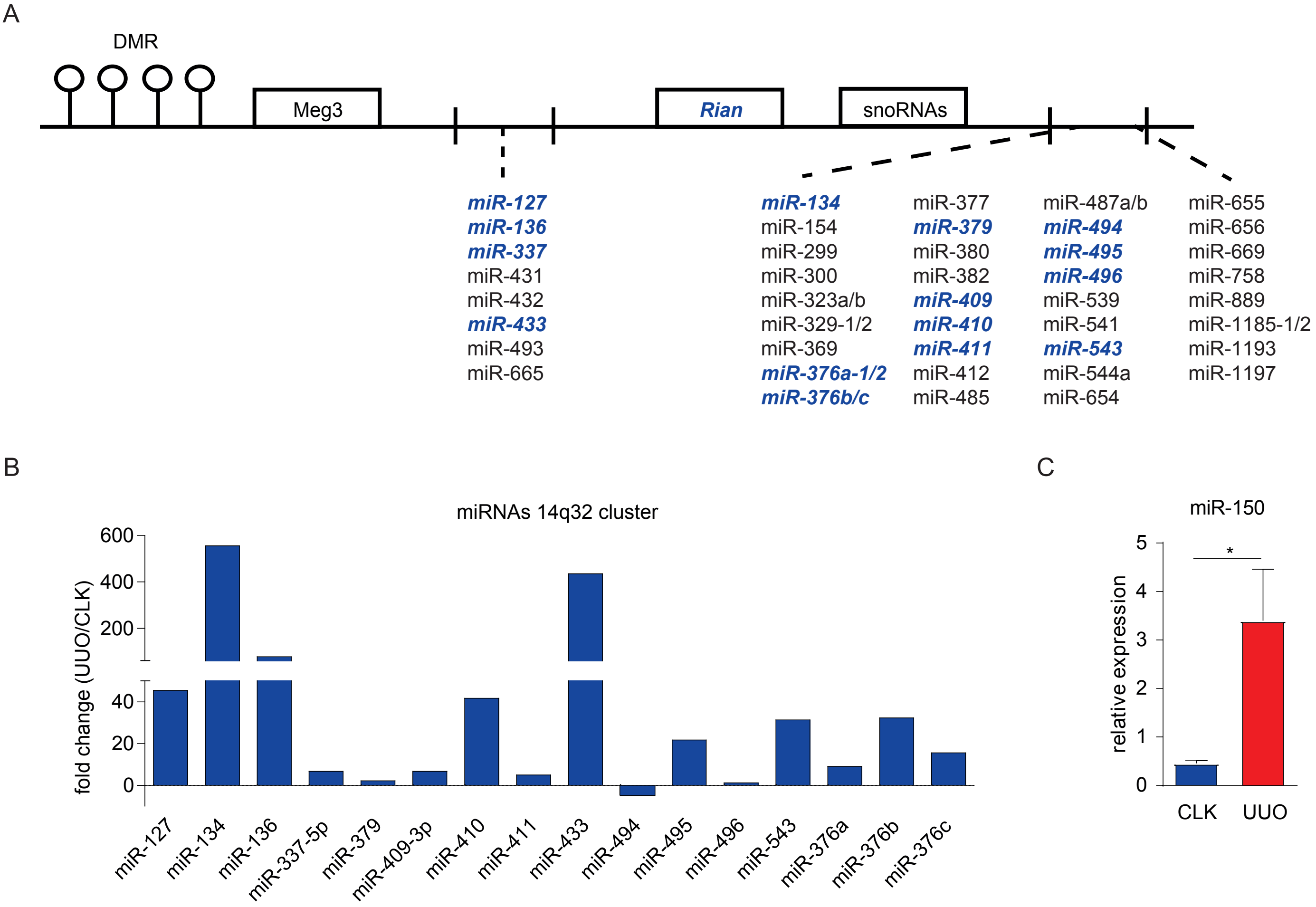

Supplementary Figure 3. Association with miRNA expression. (A) Schematic illustration of the (human) 14q32 cluster, of which the 12qF1 locus is the mouse analogue. (B) Almost all measured miRNAs located in the 14q32 cluster, where also Rian is located, show increased expression levels in the FACS sorted perivascular stromal cells from the UUO model, suggesting co-regulation of Rian and miRNAs, as this cluster has been previously identified to involve autoregulatory loops. (C) Miat-linked miR-150 is increased in FoxD1-derivative interstitial cells. *P<0.05.
